# Real-world experience with pazopanib in locally advanced and metastatic soft tissue sarcomas: a Hungarian retrospective single-center study

**DOI:** 10.3389/pore.2025.1611965

**Published:** 2025-04-01

**Authors:** Nóra Ecker, Marietta Aranyi, Edina Kiss, Nóra Kiss, Erika Lahm, Zsófia Nagy, Márta Sikter, Ádám Szabó, Anikó Szászné Szentesi, Klára Takács, Andrea Uhlyarik, József Vachaja, Barbara Sebők, Zsuzsanna Pápai

**Affiliations:** ^1^ Department of Medical Oncology, Central Hospital of Northern Pest—Military Hospital, Budapest, Hungary; ^2^ Department of Pediatric Traumatology, Dr. Manninger Jenő Accident Center, Budapest, Hungary

**Keywords:** STS, pazopanib, tyrosine-kinase inhibitor, real-world, Hungary

## Abstract

Pazopanib is a tyrosine-kinase inhibitor also used for the treatment of advanced soft tissue sarcomas. Our retrospective study analyzed real-world data of stage 4 sarcoma patients treated with pazopanib in our department in the past 10 years. Data were collected from the Medworks medical system, which is used for daily work in our center. A total of 99 patients were included: 46 men and 53 women The median age at the diagnosis was 49.8 years. The most common histological subtypes were leiomyosarcoma and synovial sarcoma. All patients received 800 mg of pazopanib per day, which was reduced to 400 mg in the event of toxicity. Treatment was continued until disease progression or unmanageable toxicity. The primary endpoint of the study was progression-free survival and the secondary endpoints were overall survival, overall response rate and disease control rate. The results in relation to demographic data, previous treatments, localizations of primary tumors and metastasis and histological subtypes were analyzed. In our center pazopanib was most frequently used in the third line. In total, 61 patients received perioperative therapy; the most common regimen used in the metastatic setting was VIP. Median PFS and OS were 3 months and 7 months, respectively. ORR was 14% and DCR was 40.45%. Dose reductions were necessary during the treatment of 56 patients. Hematological toxicity was detected in 23% of cases, with the most frequent events being grade 1 thrombocytopenia and grade 2 leukocytopenia. Non-hematological adverse events were documented in half of the patients. Pazopanib was more effective in earlier lines of treatment. Compared to the PALETTE phase 3 trial more patients received perioperative therapy, median PFS and OS were shorter (3 months vs. 4.6 months and 7 months vs. 11.9 months) and ORR was higher (14% vs. 9%) in our patient population. Dose reductions were more frequent in our center. Pazopanib is a therapeutic option for the treatment of advanced soft tissue sarcoma, also according to real-world data. Further investigations are needed to select patients who can benefit the most from pazopanib and to determine the most appropriate sequence of therapy.

## Introduction

Mesenchymal tumors are rare malignancies that account for only 1% of all adult malignancies. The incidence in Europe is 5.6/100,000 people/year [1]. Sarcoma is a broad term that includes more than 80 pathogenetically, anatomically and clinically different histological subtypes. The two major groups are soft tissue (85%) and bone (15%) sarcomas [2]. Soft tissue sarcomas can occur at any age, but their incidence is relatively higher in adolescents and adults younger than 40 years (so-called AYAs). At this age soft tissue sarcomas represent 7%-8% of all malignancies compared to 1%-2% in the overall cancer patient population [3].

The only curative strategy for the treatment of mesenchymal tumors is surgery, with or without perioperative radio- and chemotherapy [1]. Inoperable, locally advanced or metastatic cases have a poor prognosis, with a 5-year survival rate is of only approximately 16%, although there are significant differences between histological subtypes [4]. For advanced cases – with few exceptions – medical therapy is the first treatment choice, which is still traditional anthracycline-based mono- or combination chemotherapy for the majority of the patients. However, in the last few decades new alternatives (biological and immunotherapies) have appeared. Although the majority of the new modalities are only effective in some rare subtypes, pazopanib can be part of the treatment in several cases as one of the first approved and most frequently used options.

Pazopanib is a second-generation tyrosine kinase inhibitor (TKI) that targets multiple receptors, and is highly selective for inhibiting VEGFR (vascular endothelial growth factor receptor), PDGFR (platelet-derived growth factor receptor) and c-kit. By acting on these molecules the drug affects the main signaling routes of angiogenesis, tumor growth and cell survival [5–7].

In clinical trials pazopanib has shown activity in many tumor types such as renal cell carcinoma, hepatocellular carcinoma, thyroid cancer, breast cancer and soft tissue sarcoma. It was first approved for clinical use for the treatment of metastatic renal cell carcinoma [7]. The approval for metastatic soft tissue sarcoma is based on the PALETTE trial. This was a prospective double-blind placebo-controlled randomized multicenter phase 3 study; a total of 372 patients were enrolled in a 2:1 ratio. All types of adipocytic sarcoma, embryonal rhabdomyosarcoma, gastrointestinal stromal tumor, dermatofibrosarcoma protuberans and inflammatory myofibroblastic sarcoma were excluded. Median progression-free survival (PFS) was 4.6 months for pazopanib compared to 1.6 months for placebo. Overall survival (OS) was not significantly better in the pazopanib group (11.9 months) than in the placebo arm (10.4 months). The best overall response was a partial response in 6% for pazopanib and 0% for placebo, while a stable disease state was the best response in 67% for pazopanib and 38% for placebo. No complete response was documented in either group. The main adverse events were fatigue, diarrhea, nausea, weight loss, hypertension and an increase in liver enzymes. Pneumothorax occurred in a few cases. Dose reductions were necessary in 39% of patients in the pazopanib group, and the treatment was interrupted in 49%. Treatment was discontinued because of side effects in 14% of patients in the pazopanib arm [5].

In our retrospective study, we collected data from patients with advanced sarcoma who were treated with pazopanib in our department in the past 10 years. The results were analyzed in order to compare them with the PALETTE trial and to find connections between efficacy and demographics, histology, location of metastases and previous treatments. In this way, we hoped to gain a deeper understanding of the clinical behavior of these rare malignancies and to get closer to the optimal therapeutic sequences.

## Materials and methods

### Study design

Our study was a single-center retrospective data collection and analysis. We enrolled patients treated for advanced soft tissue sarcoma between 1 January 2011 and 31 December 2021 in the Oncology Department, Central Hospital of Northern Pest - Military Hospital. The aim of the study was to analyze our results with pazopanib after the progression of traditional chemotherapy in patients with locally advanced or metastatic soft tissue sarcoma and to compare them with the PALETTE trial and other real-world studies. Efficacy and toxicity were the two main focuses of our study. Clinical and laboratory data were collected at the start of the treatment, on the first day of each cycle and on day 14 of the first three cycles. Data included age, sex, histology (sarcoma subtype, grade), previous perioperative chemo- or radiotherapies, previous operations, disease-free survival after perioperative therapy, extent of disease (locally advanced or metastatic) at the start of pazopanib therapy, location of metastases, previous chemotherapy (number, types), physical and ECOG (Eastern Cooperative Oncology Group) status, laboratory findings and toxicities. CT (Computed Tomography) scans were conducted every 3 months and local MRI (Magnetic resonance imaging) examinations were also performed as needed. During the Covid period, it was decided to reduce the frequency of radiological examinations for some patients who had received pazopanib therapy for more than a year without physical signs of disease progression in order to lower the risk of infection; however, the interval between two scans was never more than 6 months. The primary endpoint of the study was progression-free survival, defined as the time from the start of pazopanib treatment to the appearance of disease progression in radiological findings, physical progression or death from any cause, whichever occurred first. Radiological progression was diagnosed by local radiologists. Tumor response was evaluated based on radiological findings and the clinical judgment of the physician.

Secondary endpoints were overall response rate (ORR), defined as the proportion of patients with complete remission (CR) or partial response (PR); disease control rate (DCR) defined as the proportion of patients with complete remission or partial response and stable disease (SD); and overall survival defined as the length of time from the start of pazopanib treatment to the death of the patient. Adverse events (AEs) were graded retrospectively according to the CTCAE (common terminology criteria for adverse events) version 4 system, based on the electronic medical records and laboratory findings.

### Study population

Our study population was selected from patients treated at our center from all over the country. Eligible patients had to meet the following inclusion criteria [1]: histologically confirmed inoperable, advanced or metastatic soft tissue sarcoma [2], pazopanib started at the standard dose of 800 mg after progression on conventional chemotherapy [3], age >18 years [4], ECOG 0-2. Patients had received at least one line of chemotherapy either in the metastatic setting or had progressed within 12 months of adjuvant treatment. Pazopanib was administered at the standard dose of 800 mg/day which was reduced to 400 mg/day in the event of toxicity. Treatment was continued until disease progression, unacceptable toxicity or death of the patient.

### Data collection and statistical analysis

Patient data were collected from the electronic medical records of the Medworks system, which is used in our hospital for daily work. In our study we analyzed the efficacy of pazopanib treatment in relation to age, sex, number of prior lines of treatment, location of primary tumor and metastases and histological subtypes. We also documented side effects and dose reductions.

The data were analyzed and entered into SPSS 25.0. Measurement data are presented as mean ± SD. Multivariate Cox regression analysis was used to identify factors influencing the prognosis of patients with advanced soft tissue sarcoma. The Kaplan-Meier method was used to plot the OS curves for these patients based on the prognostic factors, and the log-rank test was used to examine the differences in survival rates. A P value of <0.05 was considered statistically significant.

All the treated patients signed a written informed consent for pazopanib therapy as part of the pazopanib application required by the National Institute of Health Insurance Fund Management, along with consent for further use of patient data related to the disease. The study was approved by the Regional Research Ethics Committee (EPCHK-RKEB 2025/02/17-3). The study was conducted in accordance with the Declaration of Helsinki of 1964.

## Results

### Patients and baseline characteristics

**FIGURE 1:**
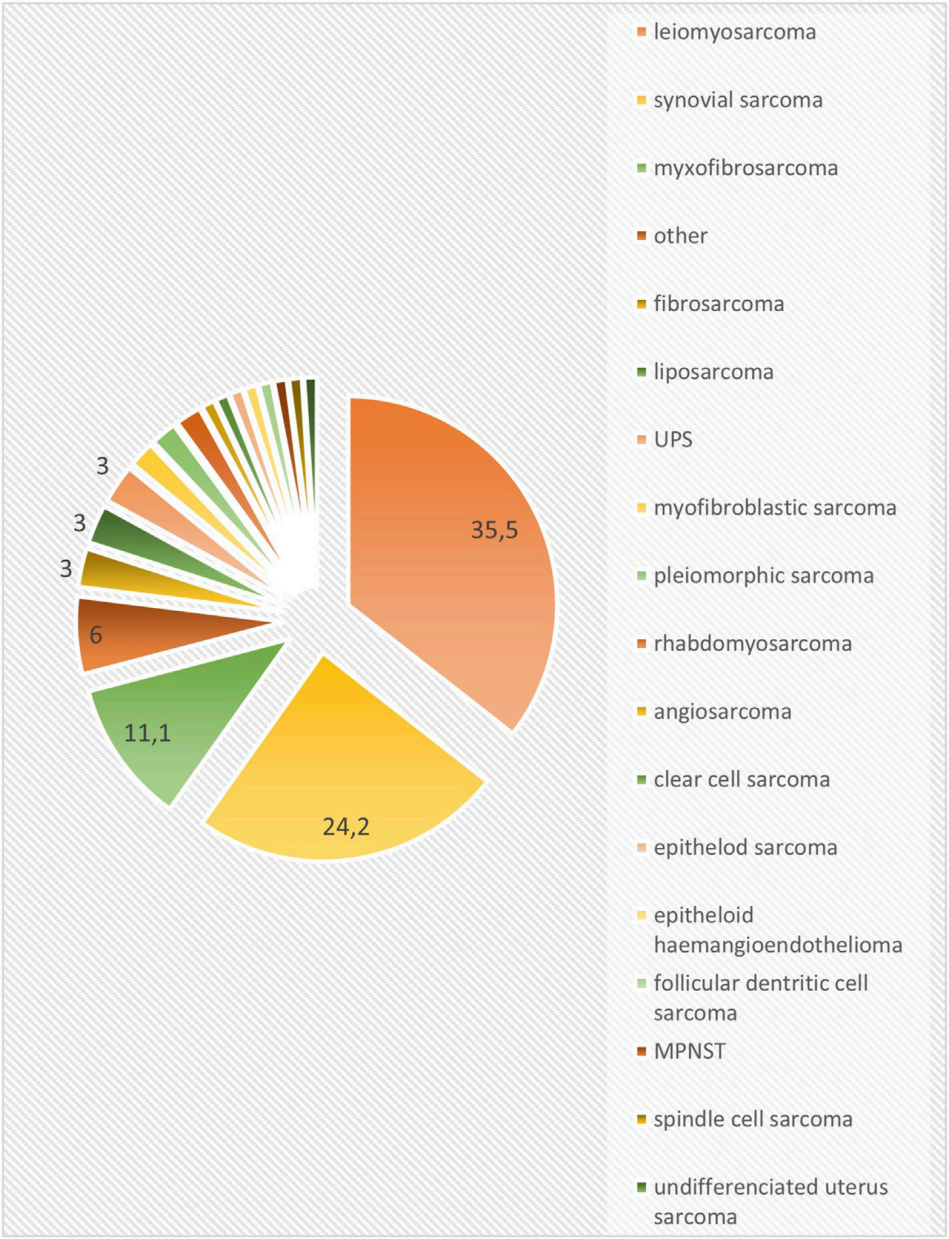
Histological subgroups (%) (UPS: undifferentiated pleiomorphic sarcoma).

A total of 99 patients were included in our retrospective data analysis. The median age at the diagnosis was 49.8 years (range: 12–86). 27.3% of our patients were adolescents or young adults (AYA, aged between 15 and 39 years); and 7% were elders (>70 years). In total, 46.5% were men and 53.5% were women. All patients were diagnosed with stage IV, inoperable, locally advanced or metastatic soft tissue sarcoma. The most common histological subtypes were leiomyosarcoma (35.5%), synovial sarcoma (24.2%) and myxofibrosarcoma (11.1%) (other subtypes are summarized in Figure 1). In total, 63.6% of patients were diagnosed with grade 3 sarcoma. The localization of the primary tumor was as follows: 48 extremity sarcomas (48.5%), 39 tumors of the trunk (39.4%), 12 retroperitoneal sarcomas (12.1%) and 1 head and neck sarcomas (1%). At the start of treatment with pazopanib, the majority of patients had pulmonary metastases (76.8%), and in 25 cases (25.3%) only the lungs were affected by the disease. A total of 30 patients (30.3%) suffered from lymph node metastases, 26 (26.3%) had hepatic metastases and 10 (10.1%) had bone metastases. Locally advanced disease without metastases was treated in 4 cases (4%) (Table 1).

**TABLE 1:** Patients’ characteristics.

Characteristics	Total (n = 99)
Age at diagnosis	
Median (years)	49.8
Range (years)	12–86
AYA	27
Adult	65
Elderly	7
Sex	
Female	53
Male	46
Grade	
1-2	14
3	63
unknown	22
Localization of primary tumor	
Extremity	48
Trunk	39
Retroperitoneal	12
Head and neck	1
Localization of metastases	
Lung	76
Only pulmonary metastases	25
Lymph node	30
Liver	26
Bone	10
Locally advanced	4

### Population

A total of 88 patients (88.9%) underwent surgery with curative intent. In total, 61 patients (61.6%) received perioperative therapy (47.5% chemotherapy alone, 9.8% radiotherapy alone, 42.6% radio- and chemotherapy) (Table 2). As for medical treatment, the majority of patients received either 4 cycles of neoadjuvant combined ifosfamide and anthracycline chemotherapy or 4 cycles of adjuvant anthracycline monotherapy. The median number of lines received from diagnosis of metastatic disease to the death of the patient was 4. Pazopanib was most frequently used as third-line treatment; a total of 15 patients (15.1%) received pazopanib as first-line therapy (after progression on perioperative anthracycline-based chemotherapy), 28 patients (28.3%) as second-line therapy, 33 patients (33.3%) as third-line therapy, 16 patients (16.1%) as fourth-line therapy, 6 patients (6%) as fifth-line therapy and 1 patient (1%) as ninth-line therapy. Before pazopanib 60 patients (60.6%) were treated with VIP (etoposide, iphosphamide, cisplatin), 25 patients (25.3%) took part in a clinical trial and 23 patients (23.2%) received DTIC-VCR (dacarbazine-vincristine) therapy. Iphosphamide-doxorubicin and gemcitabine-docetaxel were less popular regimens (Table 3). In summary, all patients except two received anthracycline-based chemotherapy before pazopanib treatment either in the perioperative or in the metastatic setting mainly in clinical trials. The median time from diagnosis of sarcoma to the start of pazopanib treatment was 20 months (range: 4–223). Before the start of pazopanib treatment 39 patients (39.4%) underwent metastasectomy and 25 patients (25.3%) received palliative radiotherapy because of symptoms or progression of a single metastasis. During pazopanib treatment 12 patients (12.1%) underwent metastasectomy and 12 (12.1%) patients received palliative radiotherapy (Table 2).

**TABLE 2:** Previous treatments.

Treatment	Total (n = 99)
Surgery	
Curative	88
Palliative	39
Radiotherapy	
Perioperative	32
Palliative	25
Chemotherapy	
Perioperative	55
Palliative	83

**TABLE 3:** Previous treatments in metastatic setting (total n = 99).

VIP	60
Clinical trial	25
DTIC-VCR	23
IFO-ADM	13
GEM-TXT	10
ADM	15
TAX	1
Other	25

VIP, etoposide, iphosphamide, cisplatin; DTIC-VCR, dacarbazine, vincristine; IFO-ADM, iphosphamide-doxorubicin; GEM-TXT, gemcitabine-docetaxel; ADM, doxorubicin; TAX, paclitaxel.

All patients started on 800 mg of pazopanib per day, which was reduced to 400 mg per day in case of toxicity. Dose reductions or temporary interruption of pazopanib were necessary during treatment in 39 patients (39.4%). In the case of 17 patients (17.2%) treatment was discontinued because of rapid clinical progression or unmanageable side effects. Hematological toxicities occurred in 23 patients (23.2%), with the most frequent being grade 1 thrombocytopenia (7%), grade 2 leukocytopenia (7%), and grade 2 anemia (5%). Non-hematological toxicity was documented in 50 patients (50%). Liver toxicity (any grade) occurred in 21 patients (21.2%), diarrhea (grade 2-3) in 21 patients (21.2%), nausea and vomiting (grade 2-3) in 15 patients (15.2%), fatigue (grade 2-3) in 14 patients (14.1%), and hypertension (grade 2-3) in 14 patients (14.1%). Pneumothorax (PTX) was documented in 2 cases, both cured in local thoracic surgery departments. No PTX-related death occurred.

In 39 patients (39.4%) pazopanib was the last active oncological therapy. After pazopanib 37 patients (37.4%) received one more treatment line, 14 patients (14.1%) received two lines and 9 patients (9%) received three or more lines.

### Clinical outcomes

**FIGURE 2:**
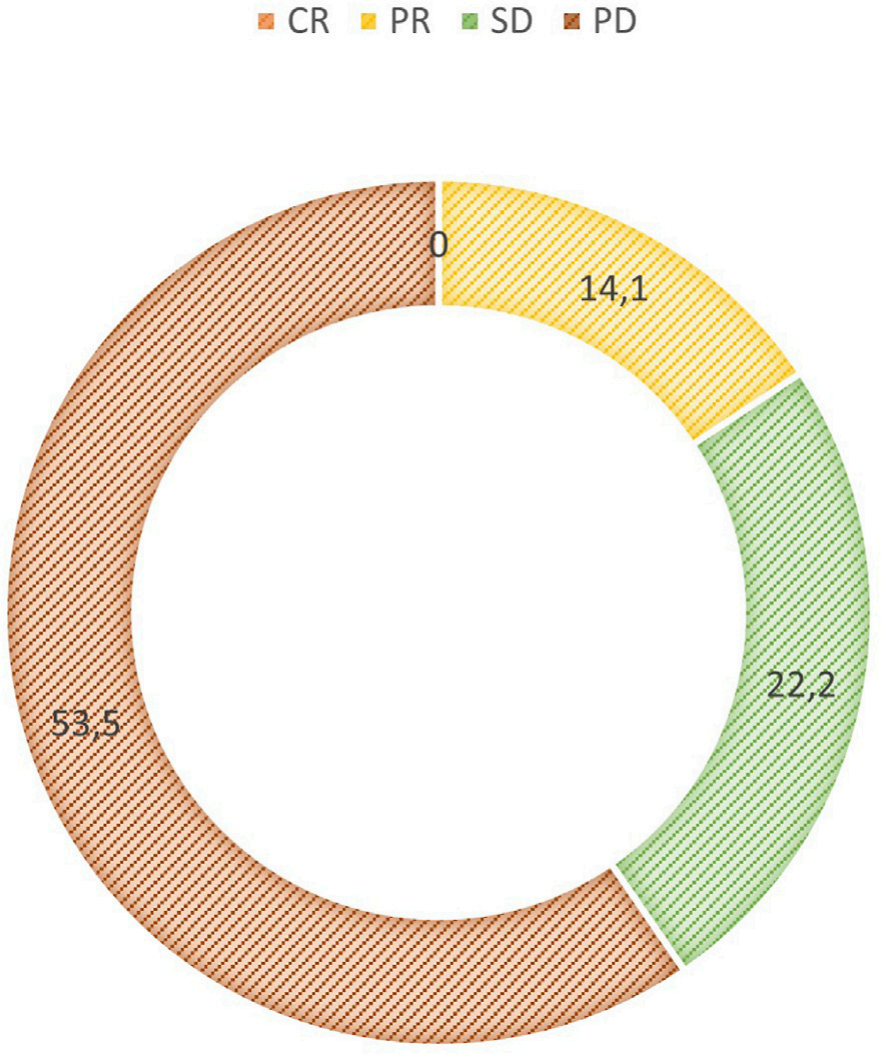
Best response CR, complete remission; PR, partial response; SD, stable disease; PD, progressive disease.

**FIGURE 3:**
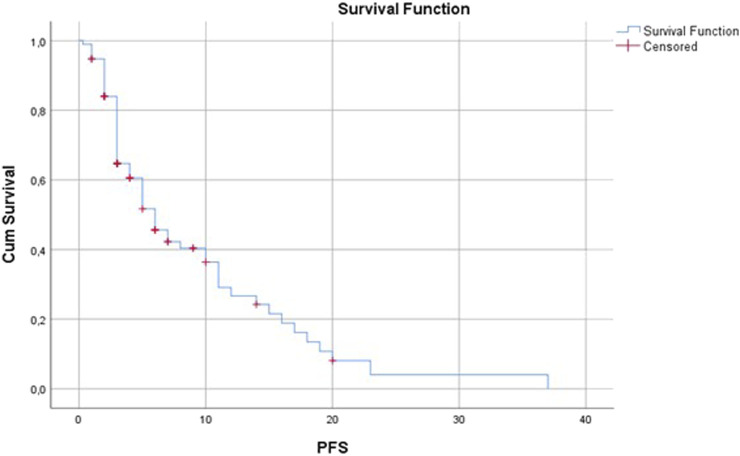
Progression-free survival (months).

**FIGURE 4:**
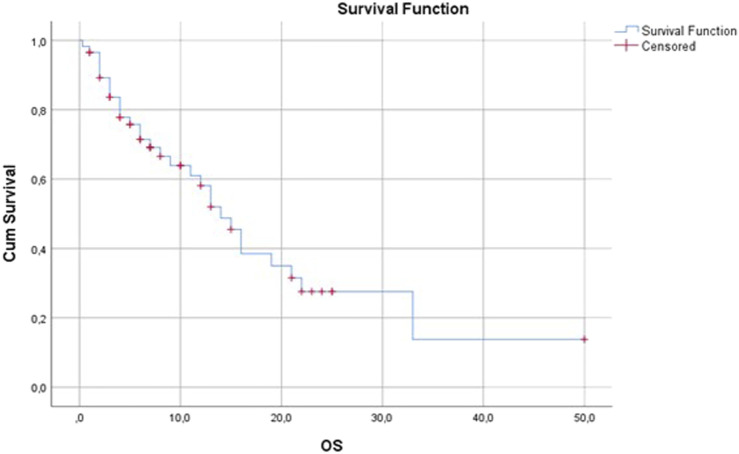
Overall survival (months).

In our patient population, the overall response rate was 14% and the disease control rate was 40.45%. No complete remission occurred. A total of 14 patients (14.1%) had a partial response, 22 patients (22.2%) had stable disease and 53 patients (53.5%) had progressive disease as the best response (Figure 2). Median progression-free survival was 3 months (range: 0.3–37) (Figure 3). Median overall survival was 17 months (range: 0.3–37) (Figure 4).

### Prognostic factors

#### Age

There were no significant differences between AYA and adult populations in terms of progression-free and overall survival. There were not enough elderly patients included in this study to analyze their data.

#### Sex

PFS and OS were not significantly different between male and female patients treated with pazopanib.

#### Histology

**FIGURE 5:**
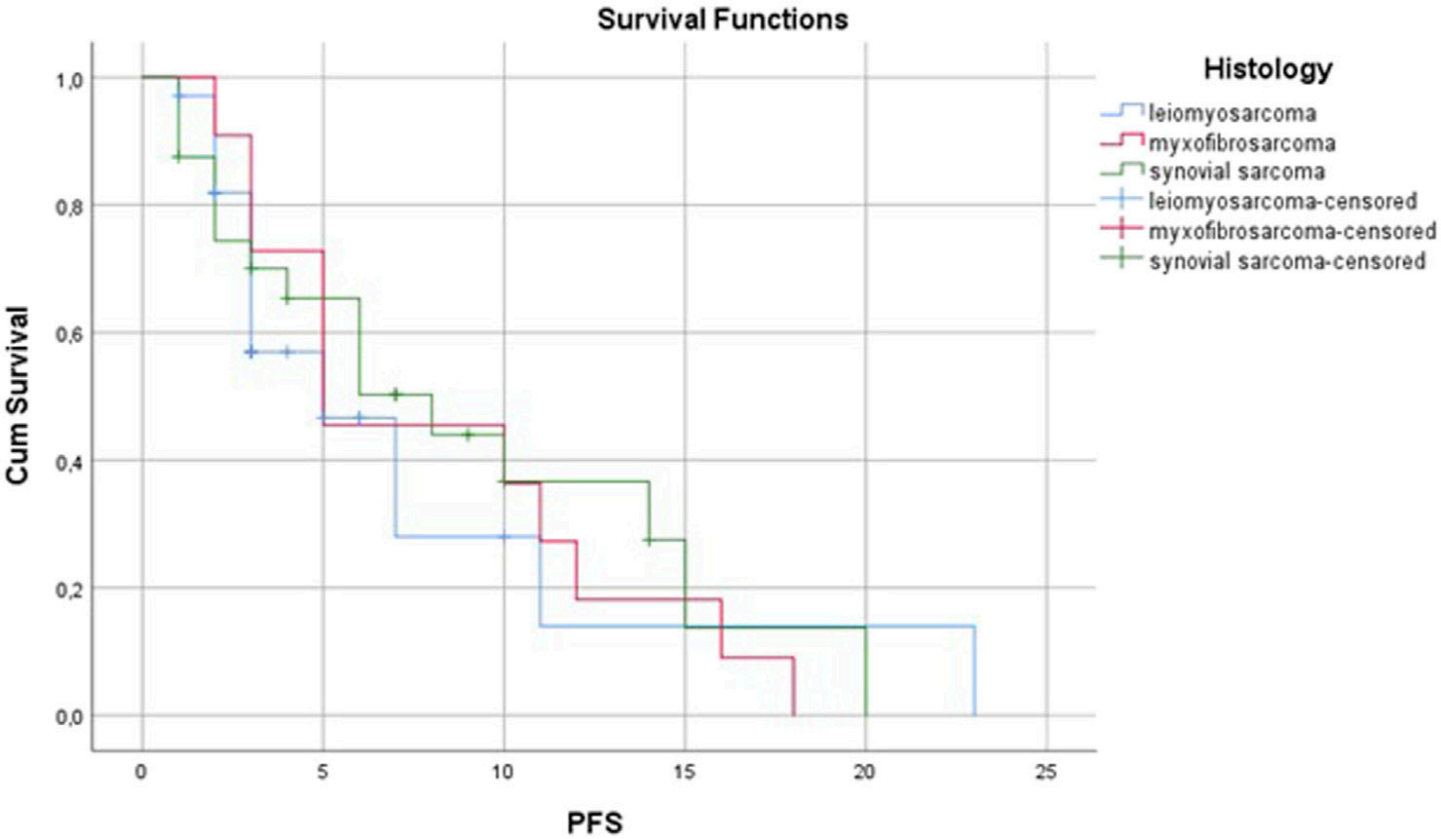
Progression-free survival by histological subtype.

Analyzing the results of the three most frequent histological subtypes we found that the most partial remissions occurred in cases of synovial sarcoma. In total, 7 of 24 synovial sarcoma patients has partial remission as best response. It was the most frequent histological subtype among patients with partial remission. Of the 36 leiomyosarcoma patients 23 (63.9%) had progressive disease (PD) as the best response, which was 43.4% of all PD cases. In myxofibrosarcoma, the overall response rate was 20% and the disease control rate was 50%. The subgroup analysis of different histological subtypes showed different progression-free survival values: leiomyosarcoma patients had the same PFS of 3 months as the whole study population, synovial sarcoma (PFS: 6 versus 3 months; p = 0.6301) and myxofibrosarcoma (PFS: 5 versus 3 months; p = 0.1108) had numerically better progression-free survival than the whole patient population, but the differences were not significant (Figure 5). There was also no difference in terms of overall survival.

#### Primary tumor location

There was no significant difference in terms of PFS and OS between trunk, extremity and retroperitoneal sarcomas.

#### Location of metastases

Based on the location of metastases, PFS and OS were slightly better in pulmonary-only metastases compared to the overall population (PFS: 4 months vs. 3 months; OS: 9 months vs. 7 months).

#### Previous treatments

**FIGURE 6:**
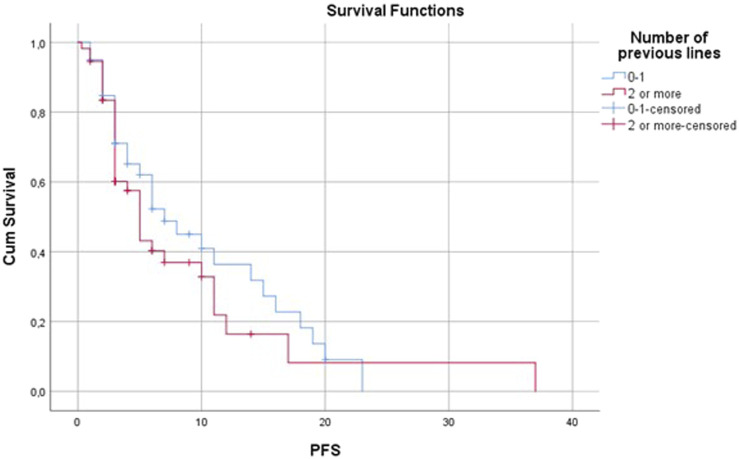
Progression-free survival by number of previous lines.

**FIGURE 7:**
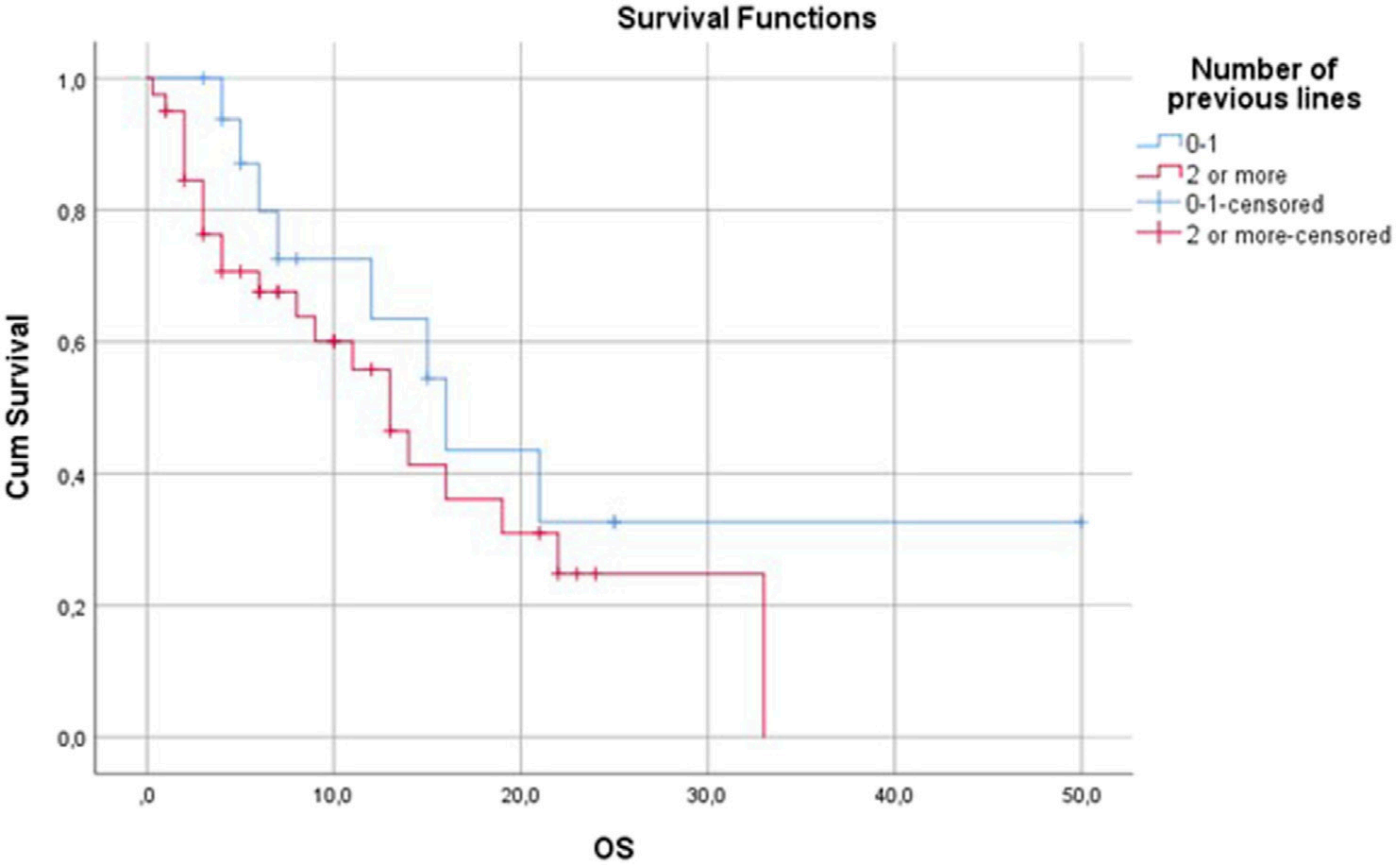
Overall survival by number of previous lines.

An improvement in disease control rate was observed when pazopanib was used in more prior lines of treatment. In the first and second lines, the DCR was 61.76%; however, in the third or more lines it was only 27.27. PFS and OS were also remarkably better in the first and second lines compared to subsequent lines (Figures 6, 7 and Table 4).

**TABLE 4:** Multivariate Cox regression equations affecting prognosis of patients.

Variables in the Equation								
	B	SE	Wald	df	Sig.	Exp (B)	95.0% CI for Exp (B)	
							Lower	Upper
Gender	−0.102	0.301	0.114	1	0.735	0.903	0.501	1.628
Histology			1,156	3	0,764			
Leiomyosarcoma	−0.239	0.421	0.323	1	0.570	0.788	0.345	1.796
Myxofibrosarcoma	0.122	0.418	0.086	1	0.769	1.130	0.498	2.563
Synovial sarcoma	0.143	0.464	0.094	1	0.759	1.153	0.464	2.863
Location of primary tumour			1.897	3	0.594			
Trunk	−0.218	1.126	0.038	1	0.846	0.804	0.089	7.299
Extremity	−0.424	0.477	0.791	1	0.374	0.654	0.257	1.667
Retroperioneum	−0.008	0.477	0.000	1	0.986	0.992	0.389	2.526
Location of metastases			1.823	2	0.402			
pulmonary	−1.266	1.061	1.426	1	0.232	0.282	0.035	2.253
multiplex	0.164	0.373	0.193	1	0.660	1.178	0.567	2.450
number of previous lines	−0.382	0.307	1.546	1	0.214	0.683	0.374	1.246

## Discussion

In this study we retrospectively analyzed the ORR, DCR, PFS and OS data of 99 metastatic sarcoma patients treated with pazopanib in our center. To our knowledge, this is the first Hungarian real-world study investigating the use of pazopanib in sarcoma. In the reviewed literature the inclusion of such a number of patients in the analog studies was rare [8].

During our experiment, the median progression-free survival was 3 months, and the overall survival was 7 months. These results are shorter than those published either in the PALETTE trial (PFS: 4.6 months, OS: 11.9 months) or in several real-world studies (RWS) [5, 8–10]. On the other hand, a few RWS reported the same PFS of approximately 3 months [11, 12]. In a meta-analysis reviewing clinical trials and real-world studies using pazopanib in second or further lines for stage 4 sarcomas, the pooled median PFS was 5.3 months [8]. Because of various prognostic factors such as heterogeneity of mesenchymal tumors (histology, grade, tumor size); different patient populations (age, sex) and discrepancies in the use of pazopanib (dose, number of previous lines, dose of previous chemotherapies) it is not easy to compare these data with our results. We believe that there are more circumstances behind the shorter survival data. First, patients with an ECOG 2 physical status or cerebral metastases who were excluded in many other RWS, and also in the PALETTE trial, were also included [5, 8]. Moreover, performance status was found to be an independent predictive factor of PFS in the EORTC phase 2 and 3 pazopanib trials [5], and the same has been reported in real-world studies [9, 13]. We believe that our patients had a worse general physical condition compared to the aforementioned study populations.

The ORR and DCR were 14% and 40.45%, respectively, according to our data. The ORR was higher and the DCR was lower than in the PALETTE trial [5]. However, in the meta-analysis of pazopanib studies an 18.9% ORR value was reported, and some RWS reported even better results. As discussed in Limitations, tumor responses were previously overestimated and the DCR was underestimated in real-world studies as a consequence of a lack of central radiological findings [8, 9, 11].

We did not measure significant differences in terms of PFS and OS between adults and AYA patients. Also, in the PALETTE trial, age was not an independent factor for PFS [5]. On the contrary, in a few real-world studies, older patients were more likely to have PD [9]. In the EORTC phase 2 and the PALETTE trials, the majority of long-term responders and survivors were young female patients with low or intermediate-grade sarcomas [5, 13, 14].

In our patient population, there was a slight majority of women, similar to the PALETTE study and other real-world trials. This may be due to the high proportion of leiomyosarcomas, which frequently arise from the uterus. Also, in the PALETTE trial, sex was not an independent prognostic factor for PFS [5].

In our study, the most frequent histological subtypes were leiomyosarcoma, synovial sarcoma and myxofibrosarcoma. These three major subtypes together accounted for more than 70% of all cases. In the majority of the literature, leiomyosarcoma is associated with better outcome data than most histological subtypes, although in the PALETTE trial histological subtype was not an independent predictive factor for PFS. However, in the PALETTE and EORTC phase 2 studies, 54% of long-term responders and survivors had leiomyosarcoma or synovial sarcoma [5, 9, 13, 14]. On the contrary the worst results were seen in leiomyosarcoma patients. As this subgroup was analyzed, it was found that leiomyosarcoma patients were more likely to receive pazopanib in later lines than patients with synovial sarcoma or myxofibrosarcoma. We suspect that the greater number of traditional chemotherapy options for leiomyosarcoma (anthracyclines, iphosphamide, gemcitabine, docetaxel, and dacarbazine) may be the reason for the later use of pazopanib. In contrast to many other sarcoma trials [9], undifferentiated pleomorphic sarcoma was quite rare in our patient population. Three patients with liposarcoma were also treated with pazopanib. In the EORTC phase 2 trial, only 25% of patients with adipocytic sarcoma treated with pazopanib were free of disease progression at 3 months [14]. Based on these preliminary data, adipocytic tumors were excluded from the PALETTE phase 3 trial [5]. However, more real-world studies and case reports with pazopanib, which included a few on patients with liposarcoma, have been published in the literature [11]. The three liposarcoma patients in our study recieved pazopanib in good general condition after all the available treatment options with the approval of the National Institute of Health Insurance Fund Management. One patient had partial remission, one had stable disease and one had progression disease as the best response to the treatment. Median PFS was 9 months and OS was 48 months. These data are of course not representative because of the low number of cases. The long OS suggests tumors with more indolent clinical behavior.

In our study population 63.6% of subjects were diagnosed with grade 3 sarcoma, and the grading was unknown in 22.2% of all cases. Only 14.2% of patients had known grade 1 or 2 tumors. In the PALETTE trial and real-world studies grade was an independent predictor of PFS [5, 9, 13, 14]. The high percentage of high-grade tumors may also be responsible for our shorter PFS and OS results.

There were no significant differences between the results of different primary tumor and metastasis locations. A Turkish study published liver metastases as a negative predictive factor [9]. IAlthough a Turkish study published liver metastases as a negative predictive factor neither primary nor metastatic tumor sites were independent predictive factors for PFS in the PALETTE trial [5, 9].

In total, 61.6% of our patients received perioperative therapy which is a remarkably high proportion compared to the PALETTE study in which only 5% of patients were treated with chemotherapy in the adjuvant or neoadjuvant setting [5]. Based on the data from the Hungarian Cancer Registry patients are frequently diagnosed with more advanced disease in Hungary [15, 16] which may explain the more frequent use of perioperative chemotherapy.

In the metastatic setting, the most frequently used regimens were VIP (etoposide, iphosphamide, cisplatin) and dacarbazine-vincristine. In the literature the therapeutic regimens used before pazopanib are not always reported, but when they are, anthracyclines and iphosphamide are the most frequently used drugs [8]. In the PALETTE study all patients had to receive anthracycline before pazopanib, and a maximum of four previous lines (only two combination regimens) were allowed [5]. The lower use of anthracycline-based chemotherapy in prior metastatic lines in our department is the result of the high proportion of perioperative chemotherapy and the ongoing clinical studies during the study period. As many patients received anthracycline-based chemotherapy in the perioperative setting, the further use of anthracyclines was not appropriate. In addition, many patients received anthracyclines in clinical trials. In summary, all patients except two received anthracycline-based chemotherapy before pazopanib treatment either in the perioperative or in the metastatic setting mainly in clinical trials. In our institute, following the national practice, VIP protocol was used after the progression of anthracycline-based chemotherapy as a substitute for high-dose iphosphamide, which was not available in Hungary during the study period. The high rate of patients with leiomyosarcoma may explain the frequent use of dacarbazine. Pazopanib was mostly administered in the third line which is the same approach as reported in the PALETTE trial [5]. In the PALETTE trial and other real-world studies, the number of previous treatment lines was an independent predictive factor for PFS and OS [5, 9, 10, 13, 14, 17]. In our study PFS was slightly longer, but DCR was higher when pazopanib was used in the first or second line.

We measured the same rate of dose reductions and treatment interruptions as in the PALETTE trial [5]. To our knowledge, there is no known direct correlation in the literature between dose reductions and pazopanib efficacy. In PK (Pharmacokinetics) studies pazopanib concentration correlates with efficacy, but as TKIs are used at fixed doses, the connection between dosage and PK concentration is uncertain [18–20]. Dosage individualization is a currently investigated issue in the field of multi-kinase inhibitors [18]. In many studies, a large proportion of patients did not receive the full standard dose or even started on a lower dose that was increased if no adverse events occurred [5, 9, 10].

In our patient population, the most frequent side effects were liver toxicity, diarrhea, nausea and vomiting, fatigue and hypertension which supports the results of the PALETTE trial. We also documented the same rate of PTX. Given the uncertainty of retrospective data collection, no further conclusions can be made.

### Limitations

The main limitation of our study is the retrospective nature of data collection. This mainly affected the assessment of non-hematological toxicities. A few adverse events had to be graded retrospectively, which decreased the accuracy of the data. Furthermore, a few patients left our department after the pazopanib treatment and continued their therapy at other centers. As a result, access to their data was not complete. Expanding our retrospective data collection to a national multicenter study may be appropriate to obtain more accurate results. Another limitation was the lack of centralized radiological analysis. The CT and MRI scans were evaluated in many radiological departments from all over the country, some of which did not use the RECIST (Response Evaluation Criteria in Solid Tumors) 1.1 criteria. This may explain the better ORR and lower stable disease rate compared to the PALETTE study. The overestimation of treatment response by physicians generally affects the results of real-world trials compared to the centralized RECIST1.1-based radiological findings of the clinical trials [8, 21].

## Conclusion

Pazopanib is a therapeutic option in metastatic sarcoma, most frequently used in earlier lines. Further investigation is needed to find the reasons for our shorter median PFS and OS data, but according to our hypothesis, the main reason could be the worse general condition of our patients and the more advanced tumor status at the time of the diagnosis. Real-world studies are important sources of information to translate the results of clinical trials into daily practice, especially in rare diseases.

## Data Availability

The raw data supporting the conclusions of this article will be made available by the authors, without undue reservation.
