# Supplementary figures and images for: Real-world experience with pazopanib in locally advanced and metastatic soft tissue sarcomas: a Hungarian retrospective single-center study
Source: Pathol Oncol Res. 2025 Apr 1;31:1611965. doi: 10.3389/pore.2025.1611965 (PMC11996657; doi:10.3389/pore.2025.1611965)

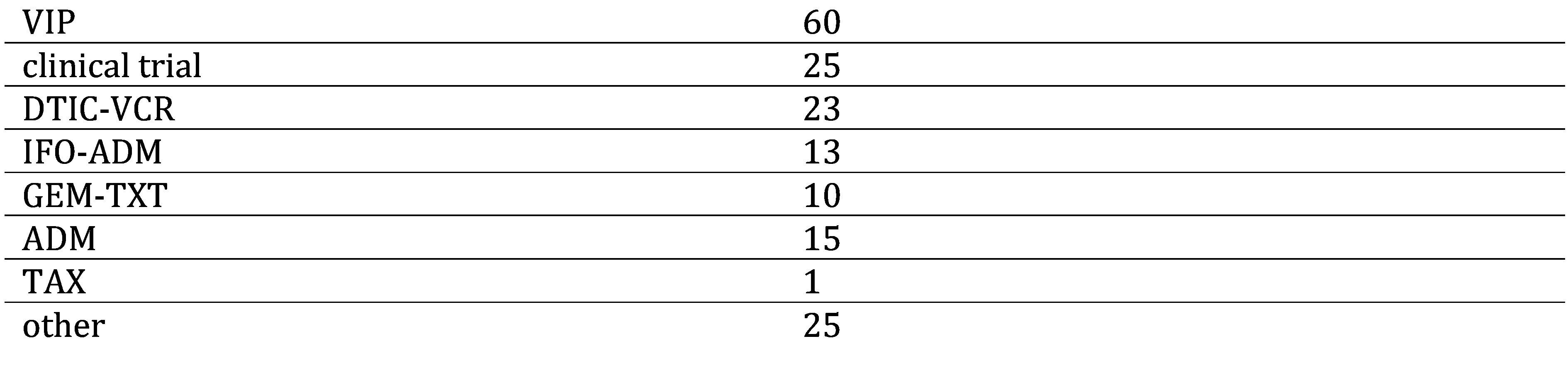

Supplement: Supplementary file 1 [file Image3.JPEG]

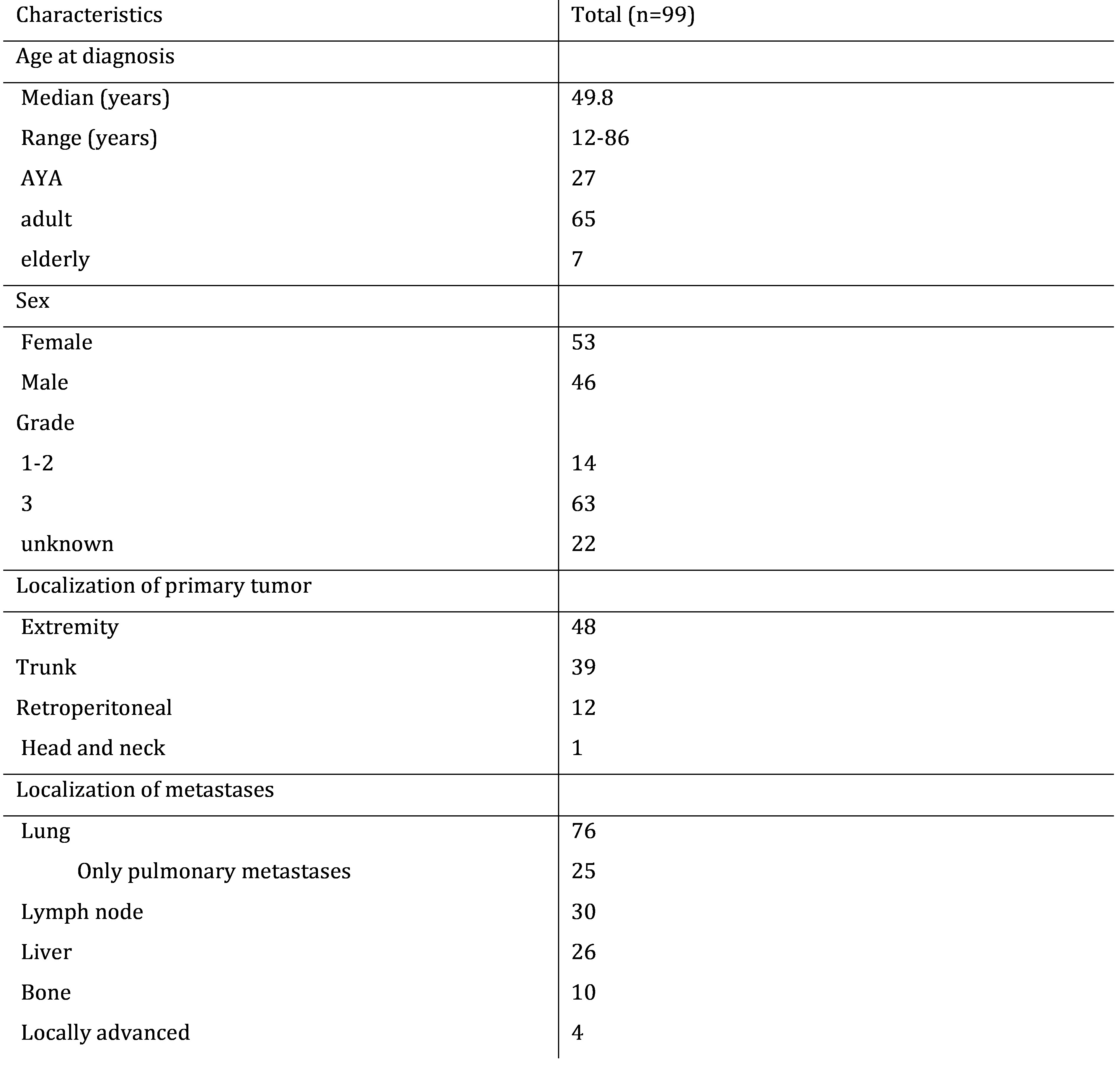

Supplement: Supplementary file 2 [file Image1.JPEG]

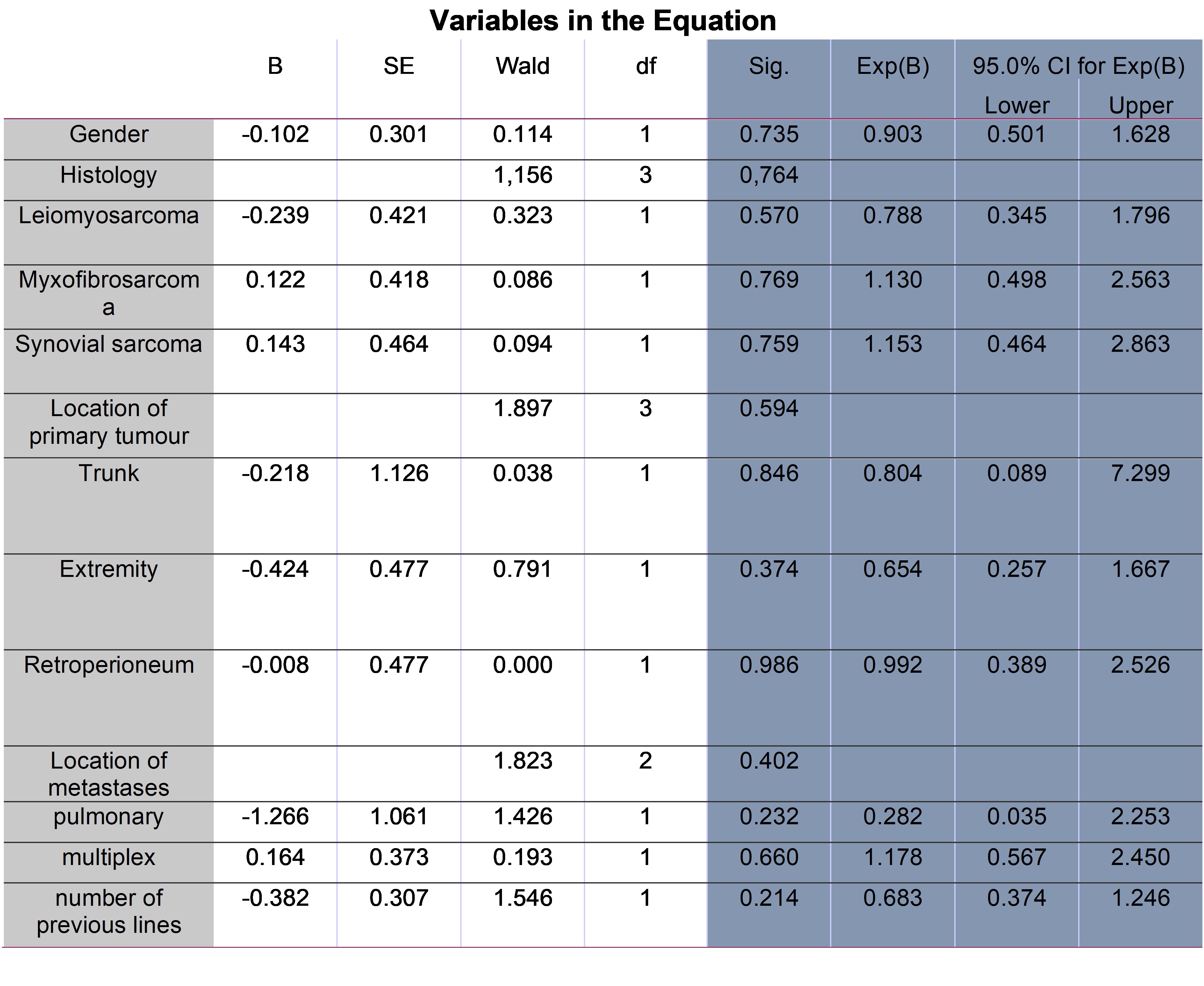

Supplement: Supplementary file 3 [file Image4.JPEG]

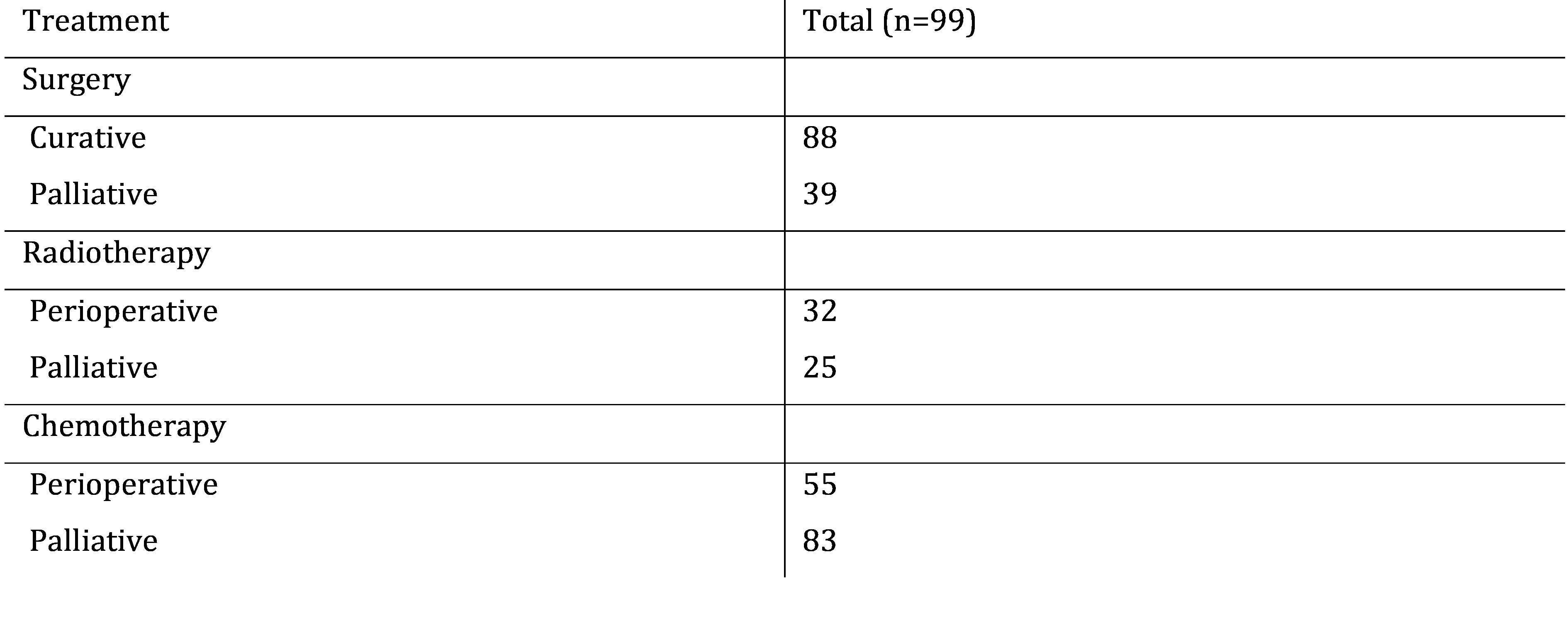

Supplement: Supplementary file 4 [file Image2.JPEG]
